# Cognitive impairment and hippocampal neuronal damage in β-thalassaemia mice

**DOI:** 10.1038/s41598-024-60459-y

**Published:** 2024-05-02

**Authors:** Nuttanan Pholngam, Parinda Jamrus, Kittikun Viwatpinyo, Benjaporn Kiatpakdee, Jim Vadolas, Pornthip Chaichompoo, Sukonthar Ngampramuan, Saovaros Svasti

**Affiliations:** 1 Graduate Program in Molecular Medicine, Faculty of Science, Mahidol University, Bangkok, Thailand; 2 Thalassemia Research Center, Institute of Molecular Biosciences, Mahidol University, Nakhon Pathom, 73170 Thailand; 3 Department of Pathobiology, Faculty of Science, Mahidol University, Bangkok, Thailand; 4 Research Center for Neuroscience, Institute of Molecular Biosciences, Mahidol University, Nakhon Pathom, 73170 Thailand; 5 Department of Medical Science, School of Medicine, Walailak University, Nakhonsithammarat, Thailand; 6 Centre for Cancer Research, Hudson Institute of Medical Research, Melbourne, Australia; 7 Department of Molecular and Translational Science, Monash University, Melbourne, Australia; 8 Department of Biochemistry, Faculty of Science, Mahidol University, Bangkok, Thailand

**Keywords:** Brain pathology, Hippocampus, Cognitive impairment, Behavior test, Aged mouse model, β-thalassaemia, Brain, Neurology, Pathogenesis, Haematological diseases, Neurological disorders

## Abstract

β-Thalassaemia is one of the most common genetic diseases worldwide. During the past few decades, life expectancy of patients has increased significantly owing to advance in medical treatments. Cognitive impairment, once has been neglected, has gradually become more documented. Cognitive impairment in β-thalassaemia patients is associated with natural history of the disease and socioeconomic factors. Herein, to determined effect of β-thalassaemia intrinsic factors, 22-month-old β-thalassaemia mouse was used as a model to assess cognitive impairment and to investigate any aberrant brain pathology in β-thalassaemia. Open field test showed that β-thalassaemia mice had decreased motor function. However, no difference of neuronal degeneration in primary motor cortex, layer 2/3 area was found. Interestingly, impaired learning and memory function accessed by a Morris water maze test was observed and correlated with a reduced number of living pyramidal neurons in hippocampus at the CA3 region in β-thalassaemia mice. Cognitive impairment in β-thalassaemia mice was significantly correlated with several intrinsic β-thalassaemic factors including iron overload, anaemia, damaged red blood cells (RBCs), phosphatidylserine (PS)-exposed RBC large extracellular vesicles (EVs) and PS-exposed medium EVs. This highlights the importance of blood transfusion and iron chelation in β-thalassaemia patients. In addition, to improve patients’ quality of life, assessment of cognitive functions should become part of routine follow-up.

## Introduction

β-Thalassaemia, arising from mutations in the β-globin gene, leads to a reduction or absence of β-globin chains in the haemoglobin molecule. This results in excess unbound α-globin chain accumulation and precipitation in erythroblasts, causing oxidative stress in erythroblasts and mature red blood cells (RBC), leading to ineffective erythropoiesis and haemolysis, respectively, and consequently chronic anaemia in patients. Multiple blood transfusions together with increased iron absorption at the gastrointestinal tract result in iron overload, which is a serious complication that plays an important role in induced systemic complications such as cardiovasculopathy, cirrhosis, multiple endocrinopathies, abnormal immunity and a shortened life expectancy^1^. Due to advances in medical treatments, the average life expectancy of patients has significantly increased. Unfortunately, the neurological complications of the patients are also gradually being recognized. Cerebrovascular disease is a well-known complication in thalassaemia patients, and a recent systematic review showed an incidence of 1.13% of cerebrovascular events in patients with β-thalassaemia, including overt ischemic stroke (0.5%), transient ischemic attack (0.23%), and silent cerebral infarcts (0.4%)^2^. In addition, magnetic resonance imaging (MRI) analysis showed a prevalence of 26.7–60.7% of silent ischemic lesions in β-thalassaemia patients^3–5^. About 36% of β-thalassaemia patients have abnormal total intelligence quotient (IQ)^6^. In addition, lower full-scale and performance IQ scores in β-thalassaemia patients have been reported^7,8^. Furthermore, about 70% of β-thalassaemia patients have mild cognitive impairment^9^. The overall risks for developing dementia were increased in thalassaemia patients^10^.

Cognitive impairment is among the most communal, troublesome, and costly disorders in aging worldwide. Cognitive impairment including delirium, mild cognitive impairment and dementia are characterized by a decline from a previously attained level of cognitive functioning^11^. In non-thalassaemia patients, lifestyle and socioeconomic factors could play a role in cognitive impairment^11^. Therefore, environmental or socioeconomic factors may also contribute to impaired cognition in β-thalassaemia patients. Several factors have been showed to correlate with cognitive impairment in β-thalassaemia patients, including age, education level, iron chelation therapy, ferritin level and haemoglobin level^9,12^.

Herein, to emphasise β-thalassaemic intrinsic factors such as anaemia, iron overload and RBC damage and to reduce confounding factors found in β-thalassaemia patients, 22- month-old β-thalassaemia mice, equivalent to about 60 years of age in humans, were used as a model in a cognitive impairment study, specifically in the domain of motor activity, and learning and memory. Furthermore, histopathology of the motor cortex and hippocampus was also examined. We found that the β-thalassaemia mice exhibited cognitive impairment that correlated with neuronal damage in the CA3 region of hippocampus, iron overload and chronic anaemia.

## Results

### Cognitive impairment in β-thalassaemia mice

**Figure 1:**
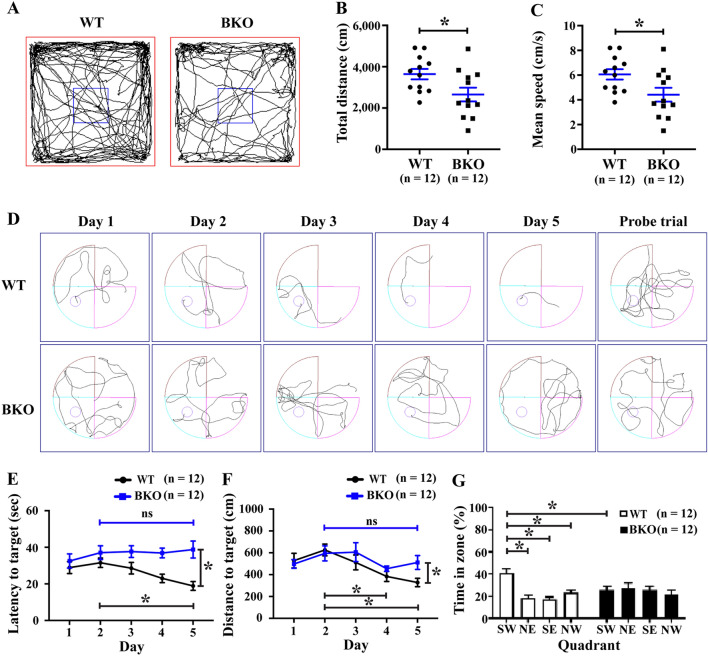
The β-thalassaemia mice had cognitive impairment. Two cognitive functions including motor activity and spatial learning and memory of 22-month-old wild type (WT) and β-thalassaemia (BKO) mice were investigated by (**A**–**C**) open field tests and (**D**–**G**) Morris water maze (MWM) tests, respectively. (**A**) Mouse trajectory path movement in the open field test. The open field test showed decreased motor activity in β-thalassaemia mice indicated by (**B**) total distance and (**C**) mean speed. Impaired spatial learning and memory of β-thalassaemia mice was elucidated by MWM tests. (**D**) Mouse swimming tracks in the MWM tests. The learning curve of mice during the learning phase in the MWM was assessed for (**E**) latency to find the target platform and (**F**) distance to the target platform. **(G)** Percentages of time spent in the target quadrant at probe trial test. Data presents as mean ± SEM. *Statistically significant difference between groups at *P* < 0.05. *ns* not significant difference between groups.

Cognitive function, especially motor activity, learning and memory in 22-month-old, β-thalassaemia mice was investigated by open field and Morris water maze (MWM) tests, respectively. The open field test shows the free exploration and locomotion ability in a new environment. Decreased total distance and mean speed of movement in the β-thalassaemia mice was observed (Fig. 1A–C), suggesting that the β-thalassaemia mice have reduced motor activity when compared with wild type mice.

Spatial learning and memory impairment was ascertained by a MWM test, based on the principle that mice can learn to navigate spatial environments using external cues (Fig. 1D–G). During the 5-days platform learning period, with increase of training times, wild type mice show significantly decreased latency time and distance to find the platform (Fig. 1E,F). The latency to target on day 5 (18.8 ± 2.4 s) was about 40% faster than that on day 2 (31.6 ± 2.6 s), while distance to target on day 5 (328.1 ± 38.7 cm) was about 50% shorter than that on day 2 (626.0 ± 53.9 cm). In contrast, the β-thalassaemia mice showed no decreased latency and distance to target between day 5 and day 2. Spatial memory was tested by removing the platform in a probe trial. Wild type mice spent significantly increased time in the platform quadrant, (40.97 ± 3.8%, Fig. 1G), while there was no difference in the target-quadrant duration (25.49 ± 3.3%) compared to other quadrant zones with the β-thalassaemia mice. Together, these findings indicate β-thalassaemia mice have spatial memory impairment compared to age-matched wild type mice.

### Increased hippocampus neuronal damages in β-thalassaemia mice

**Figure 2:**
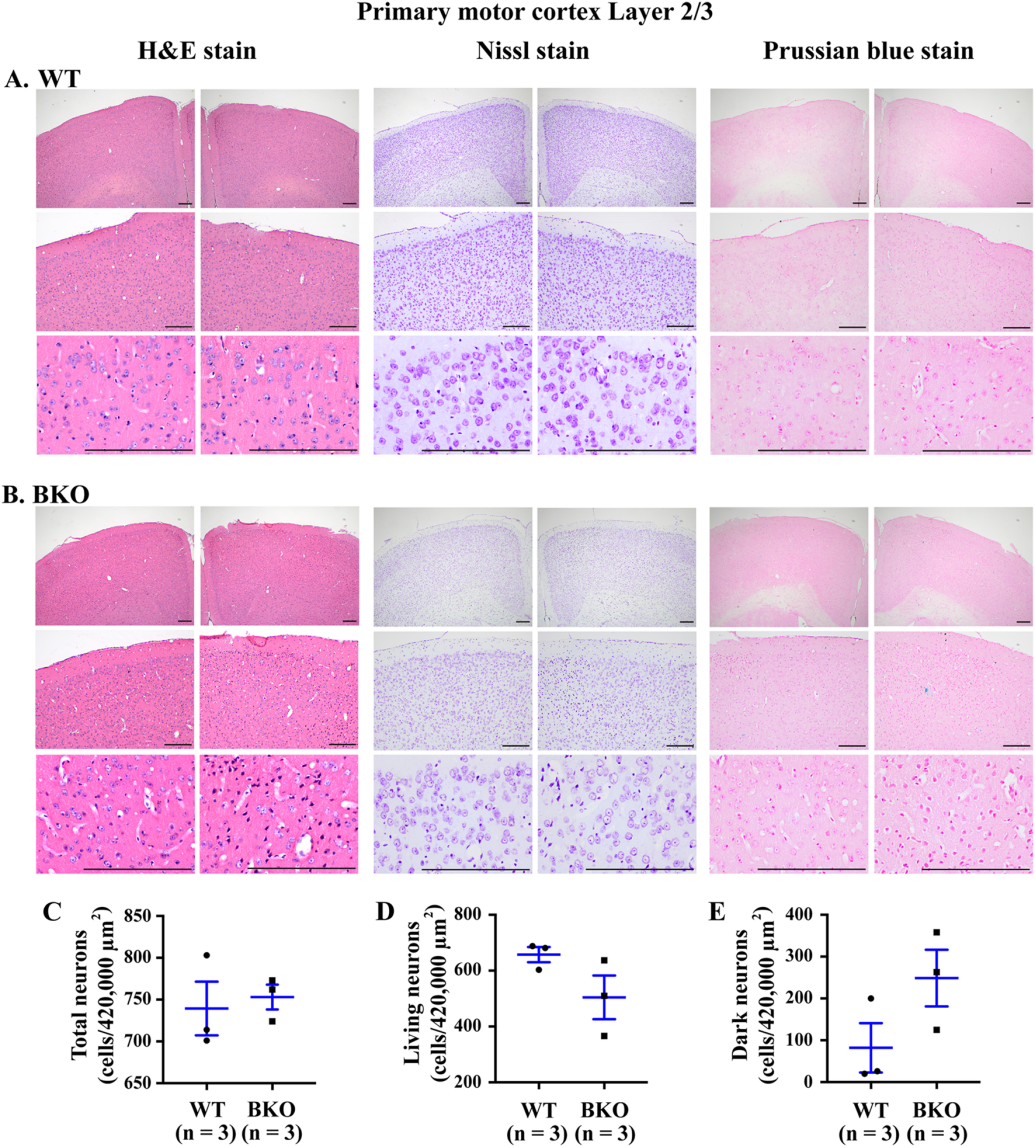
No neuronal degeneration in primary motor cortex, layer 2/3 of β-thalassaemia mice. Brains from (**A**) 22-month-old wild type (WT) mice and (**B**) 22-month-old β-thalassaemia (BKO) mice were stained with haematoxylin and eosin (H&E) for anatomical analysis (left panel), Nissl stain for counting living and dark neuron cells (middle panel) and Perls’ Prussian blue stain for the evaluation of iron deposition (right panel). Scale bar is 200 μm. (**C**) Total neuron cells, (**D**) living neurons and (**E**) dark neuron in primary motor cortex, layer 2/3 from left- and right-side of brain were counted and identified using Nissl stain (Supplementary Figs. S3, S4). Data presents as mean ± SEM.

The primary motor cortex is the supraspinal motor control centre that directly communicates with most of the other motor control structures. To assess whether the reduced locomotor activity in open field test relates to motor cortex damage in β-thalassaemia mice, the morphology of the primary motor cortex area for layer 2/3 (MOp layer 2/3) was examined. No haemorrhage, inflammation or necrosis in both β-thalassaemia and wild type mice was observed after haematoxylin and eosin staining (Fig. 2A,B). The primary motor neuron was then examined by Nissl staining. Total neurons in MOp layer 2/3 did not significantly differ in β-thalassaemia mice compared to wild type mice (Fig. 2C). In addition, normal living neurons and dark neurons (a hallmark of damaged neurons) which are defined as shrunken and condensed chromatic-hyperchromatic nuclei within the cell body, were also not significantly different between groups (Fig. 2D–E). Iron overload is a major complication in β-thalassaemia. Furthermore, brain iron deposit has been reported to be associate with cognitive impairment. Evaluating iron deposition by Perls’ Prussian blue staining was performed and showed no iron deposition in MOp layer 2/3 (Fig. 2A,B).

**Figure 3:**
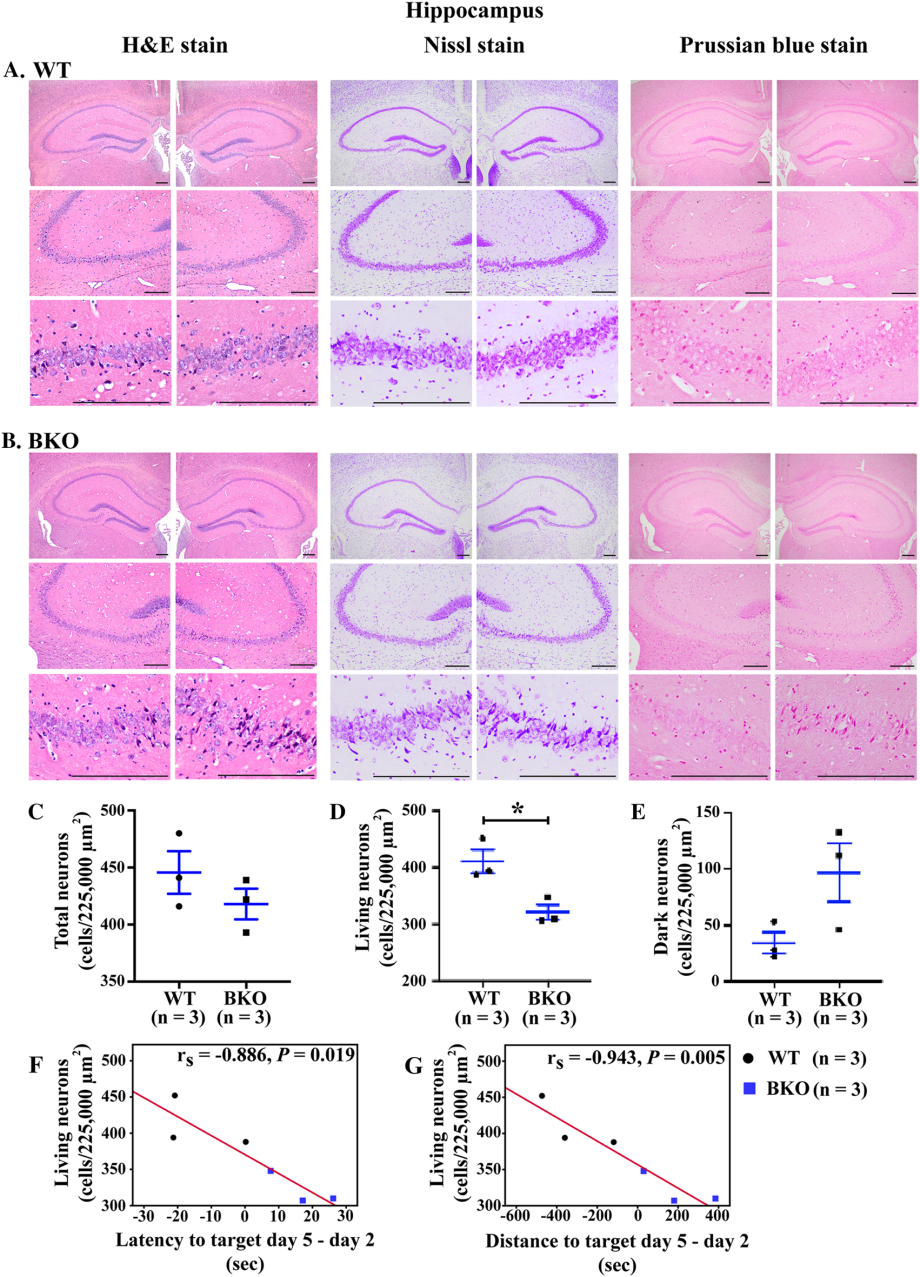
Decreased living pyramidal cells in hippocampal CA3 region of β-thalassaemia mice. Histopathological analysis of the hippocampus from (**A**) 22-month-old wild type (WT) mice and (**B**) 22-month-old β-thalassaemia (BKO) mice stained with haematoxylin and eosin (H&E) for anatomical analysis (left panel), Nissl staining for counting living and dark neurons (middle panel) and Perls’ Prussian blue staining for the evaluation of iron deposition (right panel). Scale bar is 200 μm. The β-thalassaemia mice have decreased living pyramidal cells in hippocampal CA3 region. (**C**) Total pyramidal neurons, (**D**) living neurons and (**E**) dark neurons in the hippocampal CA3 region from left- and right-side of the brain were counted and identified as living and dark neurons using Nissl stain (Supplementary Figs. S5–S7). The living pyramidal cells in the hippocampal CA3 region is correlated with cognitive function. Spearman’s rho correlation coefficient analysis between living pyramidal cells in the hippocampal CA3 region and (**F**) difference time of latency to target between day 5 and day 2 and (**G**) difference of distance to target between day 5 and day 2. Data presented as mean ± SEM. *Statistically significant difference between groups at *P* < 0.05.

The hippocampus is an important encephalic region related to learning and memory, and the hippocampus converts short-term memory into long-term memory, solves spatial memory problem and recollects past experiences of places. To explore whether learning and memory impairment are associated with degenerated damaged neurons in the hippocampal area of β-thalassaemia mice, structure and neuronal damage in the hippocampus were examined. Histological analysis of hippocampal formation using haematoxylin and eosin demonstrated no haemorrhage, inflammation or necrosis in both β-thalassaemia and age-matched wild type mice (Fig. 3A,B). The CA3 region of hippocampus is well-recognized for its special role in memory processes. Therefore, a Nissl stain was performed and analysed the pyramidal cells at CA3 region (Fig. 3C–E). It is noteworthy that the living neuron number was significantly reduced in β-thalassaemia mice as compared to wild type mice (Fig. 3D). Importantly, the spatial learning MWM performance was correlated with living neurons number at the CA3 region (Fig. 3F,G). The difference between day 5 and day 2 (Δday 5- day 2) performance of both latency and distance to target correlated with living neuron numbers. Interestingly, there was no iron deposition in hippocampus of β-thalassaemia mice as demonstrated by Perls’ Prussian blue staining (Fig. 3A,B).

### Cognitive impairment in β-thalassaemia mice correlated with tissue iron, anaemia and RBC damage

**Figure 4:**
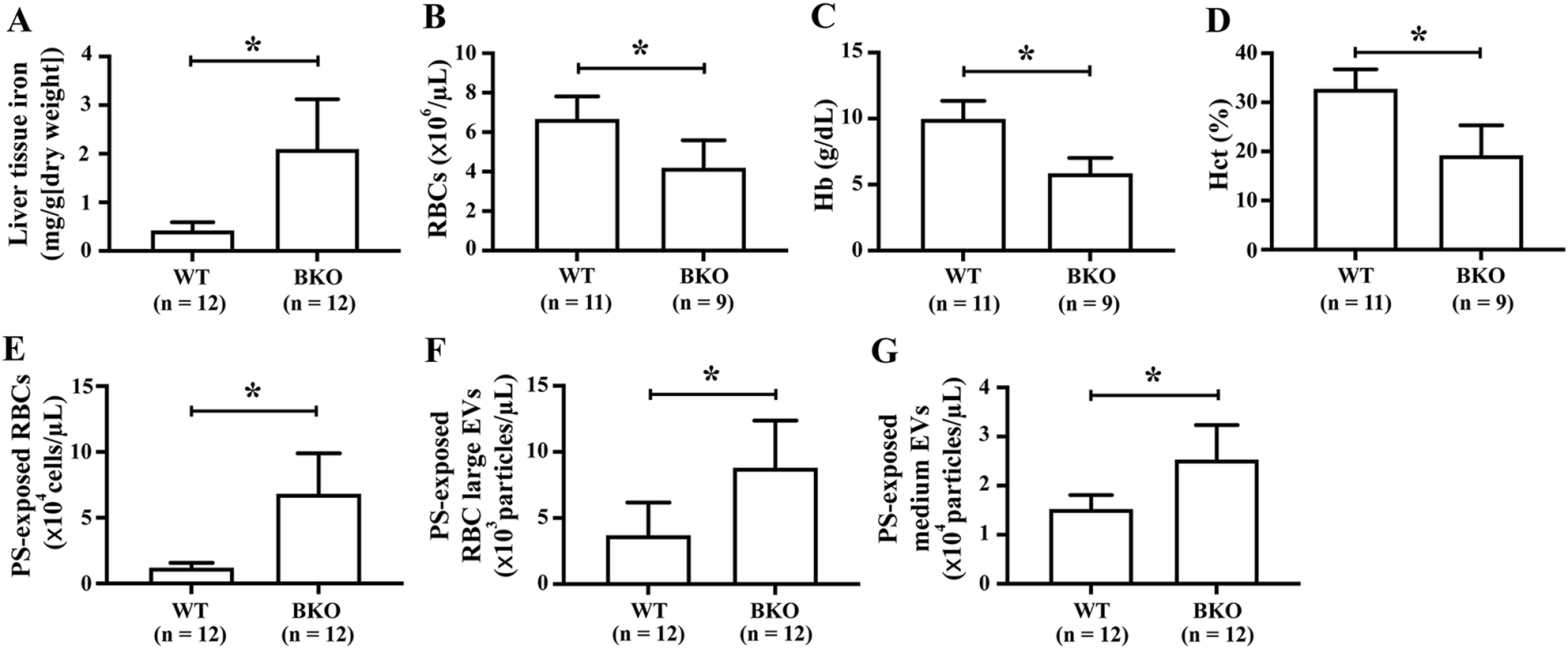
Clinical pathology of β-thalassaemia mice. β-Thalassaemia (BKO) and wild type (WT) mice were evaluated for (**A**) liver tissue iron and anaemia markers including (**B**) red blood cell (RBC) count, (**C**) haemoglobin (Hb) and (**D**) haematocrit (Hct); and damaged RBC index including (**E**) phosphatidylserine (PS)-exposed RBCs, (**F**) PS-exposed RBC large extracellular vesicles (EVs) and (**G**) PS-exposed medium EVs. Data presents as mean ± SD. *Statistically significant difference between groups at *P* < 0.05.

**Figure 5:**
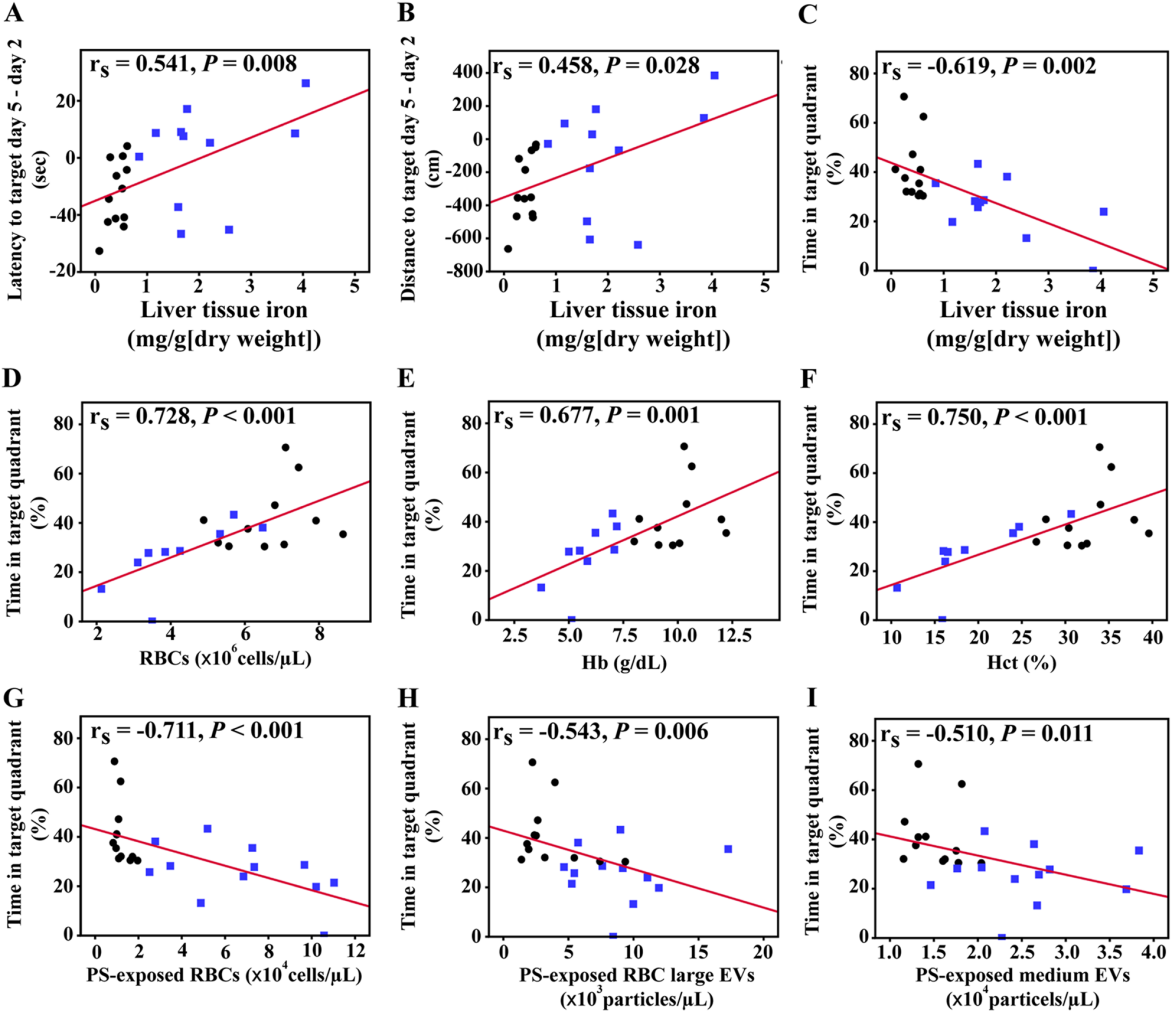
Cognitive impartment correlated with iron overload, anaemia and RBC damage in β-thalassaemia mice. Spearman’s rho correlation coefficient was used to analysed the relationship between liver tissue iron and behavior in spatial learning and memory tests using the MWM test including (**A**) different time of latency to target between day 5 and day 2, (**B**) different distance to target between day 5 and day 2, and (**C**) percentages of time in target quadrant. Percentages of time in target quadrant were correlated with (**D**) RBC count, (**E**) haemoglobin (Hb), (**F**) haematocrit (Hct), (**G**) phosphatidylserine (PS)-exposed RBCs, (**H**) PS-exposed RBC large extracellular vesicles (EVs) and (**I**) PS-exposed medium EVs.

Cognitive impairment in β-thalassaemia patients has been showed to correlate with socio-economic status, age, iron chelation therapy, ferritin level and haemoglobin level^9,12^. Herein, thalassaemia intrinsic factors including iron overload and anaemia were determined in β-thalassaemia mice. Increased liver tissue iron level and decreased RBC number, haemoglobin andhaematocrit, the hallmarks of anaemia were observed in β-thalassaemia mice (Fig. 4A–D). To investigate the contribution of the thalassaemia intrinsic factors to cognitive impairment, the correlation between learning and memory impairment parameters with liver tissue iron and anaemia was assessed by Spearman’s rho correlation coefficient analysis. A significant correlation was observed between liver iron levels and learning and memory impairment parameters including difference of latency to target on day 5 and day 2, difference of distance to target on day 5 and day 2 and time in target quadrant (Fig. 5A–C). In addition, time spent in the target quadrant was correlated with anaemia markers including RBC number, haemoglobin and haematocrit (Fig. 5D–F).

Learning and memory impairment based on haemoglobin and liver iron levels was then explored. In this study, learning and memory impairment was defined as values representing the percentage of time in the target quadrant of wild-type mice that were lower than the mean minus 1 standard deviation, specifically 27.83%. The predictability of haemoglobin levels 5.5–6.5 g/dL and liver iron level 1.0–2.0 mg/g was analysed (Table 1). Haemoglobin level cutoff at ≤ 6.0 g/dL yielded the high accuracy, 88.9%, sensitivity, 100.0%, and specificity, 80%. While liver iron level cutoff at ≥ 1.80 mg/g resulted in the high accuracy, 77.8%, sensitivity, 75.0%, and specificity, 80%. The combination of both haemoglobin level at ≤ 6.5 g/dL and liver iron level at ≥ 1.6 mg/g yielded the highest accuracy, 100%, sensitivity, 100%, and specificity, 100%. This emphasises the impact of iron overload and anaemia on cognitive impairment in β-thalassaemia.

**Table 1:** Prediction of learning and memory impairment based on haemoglobin and liver iron levels.

Hb	LIC	Sensitvity (%)	Specificity (%)	Accuracy (%)	OR	95% CI	*p*-value
≤ 5.5	–	75.00	80.00	77.78	12.00	0.51–280.11	0.099
≤ 6.0	**–**	100.00	80.00	88.89	NA	NA	NA
≤ 6.5	–	100.00	60.00	77.78	NA	NA	NA
–	≥ 1.00	100.00	20.00	55.56	NA	NA	NA
–	≥ 1.20	100.00	20.00	55.56	NA	NA	NA
–	≥ 1.40	100.00	20.00	55.56	NA	NA	NA
–	≥ 1.60	100.00	40.00	66.67	NA	NA	NA
**–**	≥ 1.80	75.00	80.00	77.78	12.00	0.51–280.11	0.099
–	≥ 2.00	75.00	80.00	77.78	12.00	0.51–280.11	0.099
≤ 5.5	≥ 1.00	75.00	80.00	77.78	12.00	0.51–280.11	0.099
≤ 5.5	≥ 1.20	75.00	80.00	77.78	12.00	0.51–280.11	0.099
≤ 5.5	≥ 1.40	75.00	80.00	77.78	12.00	0.51–280.11	0.099
≤ 5.5	≥ 1.60	75.00	100.00	88.89	NA	NA	NA
≤ 5.5	≥ 1.80	50.00	100.00	77.78	NA	NA	NA
≤ 5.5	≥ 2.00	50.00	100.00	77.78	NA	NA	NA
≤ 6.0	≥ 1.00	100.00	80.00	88.89	NA	NA	NA
≤ 6.0	≥ 1.20	100.00	80.00	88.89	NA	NA	NA
≤ 6.0	≥ 1.40	100.00	80.00	88.89	NA	NA	NA
≤ 6.0	≥ 1.60	100.00	100.00	100.00	NA	NA	NA
≤ 6.0	≥ 1.80	75.00	100.00	88.89	NA	NA	NA
≤ 6.0	≥ 2.00	75.00	100.00	88.89	NA	NA	NA
≤ 6.5	≥ 1.00	100.00	80.00	88.89	NA	NA	NA
≤ 6.5	≥ 1.20	100.00	80.00	88.89	NA	NA	NA
≤ 6.5	≥ 1.40	100.00	80.00	88.89	NA	NA	NA
≤ 6.5	≥ 1.60	100.00	100.00	100.00	NA	NA	NA
≤ 6.5	≥ 1.80	75.00	100.00	88.89	NA	NA	NA
≤ 6.5	≥ 2.00	75.00	100.00	88.89	NA	NA	NA

*Hb* haemoglobin (g/dL), *LIC* liver iron content (mg/g (dry weight)), *OR* odd ratio, *NA* not available.

Another hallmark of β-thalassaemia is damaging RBCs due to increased oxidative stress and iron overload, which consequently increases RBC membrane damage, indicated by increased PS-exposed RBCs. In addition, due to increased RBC membrane damage, increased vesiculation of PS-exposed RBC large EVs and PS-exposed medium EVs is observed in the blood circulation of β-thalassaemia patients and β-thalassaemia mouse^13,14^. Increased RBC damage in β-thalassaemia mice was demonstrated by increased PS-exposed RBCs, PS-exposed RBC large EVs and PS-exposed medium EVs (Fig. 4E–G). An association between liver iron levels and RBC damage markers was found (Supplementary Fig. S1). Interestingly, time spent in the platform quadrant was correlated with levels of PS-exposed RBC_S,_ PS-exposed RBC large EVs and PS-exposed medium EVs (Fig. 5G–I).

## Discussion

As the life expectancy of the patients has increased, cognitive impairment has been become more recognized. In non-thalassaemia patients, in addition to intrinsic factors, lifestyle and socioeconomic factors could play a role in cognitive impairment^11^. Furthermore, an association between education and cognitive impairment in β-thalassaemia has been reported^9^. Herein, to eliminate confounding socioeconomic effects, 22-month-old β-thalassaemia mice were used as a model to investigate thalassaemic intrinsic factors, including anaemia, iron overload and RBC damage, on cognitive function in β-thalassaemia disease.

The β-thalassaemia mice exhibiting cognitive impairment resemble β-thalassaemia patients. A Montreal Cognitive Assessment (MoCA) of β-thalassaemia patients at age 35 ± 11 years showed a high prevalence (70.2%) of mild cognitive impairment^9^. A Wechsler Adult Intelligence Scale-Fourth Edition (WAIS-IV) test of β-thalassaemia patients at age 34.5 ± 10.3 years showed increased cognitive impairment in explored learning memory, perceptual-motor, and executive function^8^. Another study documented neuropsychological impairment involving multiple cognitive domains including exploring abstract reasoning, attention and psychomotor speed, executive functions, language, constructional and visuospatial skills, and short- and long-term verbal and visuospatial memory in β-thalassaemia patients at age 25.3 ± 6.1 years^15^. Furthermore, thalassaemia patients aged over 65 years were 2.24-fold more likely to develop dementia (95% CI 1.17–4.29) compared with the control cohort aged over 65 years^10^.

The β-thalassaemia mice have impaired motor activity indicated by reduced total distance movement and mean speed as compared to the age matched wild type mice. However, morphological markers of neuronal damage in the primary motor cortex area did not differ between groups. The primary motor cortex is the primary region of the motor cortex system to control the execution of movement and to integrate motor signals to both cortical and subcortical areas^16^. The interaction between cognitive and motor functions is the complexity that overlaps in the motor cortex and the other brain areas, such as cerebellum, basal ganglia, premotor cortex, thalamus, and precuneus^17^. Therefore, impaired motor activity in β-thalassaemia mice might arise from abnormality in other brain areas.

The β-thalassaemia mice were markedly deficient in spatial learning and memory, as indicated by no decrease in latency time and distance over successive trials and no increase in time spent in the target quadrant during the probe trial compared to age-matched wild type mice. Although β-thalassaemia mice exhibited impaired locomotor activity, this impairment in locomotor activity is dissociated from learning memory impairment as swim speeds in MWM were reported to be unaffected by decreases in land-based locomotion^18^. The β-thalassaemia mice have reduced living neuron numbers in the hippocampus, which correlated with the spatial learning and memory tests. The hippocampal neuron loss is correlated with cognitive deficits in spontaneous age-related neurodegenerative changes in mice^19^. Interestingly, β-thalassaemia mice showed accelerated neurodegenerative alteration which may be cause by iron accumulation, chronic anaemia, and RBC damage.

Abnormal accumulation of brain iron has been detected in various neurodegenerative diseases^20^. Intriguingly, our study revealed a correlation between iron overload and cognitive dysfunction, consistent with the study in β-thalassaemia patients. Impaired cognition in the patients is associated with a lack of iron chelation therapy, serum ferritin levels and haemosiderosis^9,12,15^. However, the iron content in the brain of β-thalassaemia patients is still debatable. MRI studies in β-thalassemia patients have reported brain iron accumulation in various regions, such as the motor and temporal cortex, putamen, caudate nucleus, and basal ganglia^21,22^. In contrast, a recent MRI study on brain iron content in β-thalassaemia patients showed iron accumulation in the brain unambiguously confined to the choroid plexuses^23^. In addition, MRI assessments found no correlation between hippocampal iron content and hippocampal volume, and no significant difference in hippocampal volume between β-thalassemia patients and controls was observed^23^. Interestingly, brain tissue iron content, quantitatively assessed by MRI, did not correlate with lower full-scale IQ or any WAIS subscore^8^. In this study, Perls’ Prussian blue staining found no increase in iron accumulation in the hippocampus of β-thalassaemia mice, consistent with a previous study that revealed a slight deposition of iron in microglial cells in the cerebral cortex^24^. Although brain iron overload has been indicated as possible mechanisms of brain injury due to its potential neurotoxicity, it is noteworthy that this study showed no neural tissue iron overload by Perls’ Prussian blue staining. It seems that iron overload severely affects several organs but not brain. This may be caused by the tight control of iron distribution in the brain by astrocytes, which release hepcidin to diminish brain iron uptake at blood–brain barrier (BBB) via regulating ferroportin 1^25^. In addition, astrocytes and microglia regulate excessive iron in the brain^26,27^.

There is no report about impairment of the BBB in β-thalassaemia patients. The systemic iron overload in β-thalassaemia mice could induce a dysfunctional BBB, allowing more iron or other cytotoxic molecules to enter the brain. Intracellular Fe^2+^ accumulation in brain microvascular endothelial cells and pericytes leads to BBB dysfunction^28^. The excessive iron generates free radicals that accumulate in cerebral endothelial cells, contributing to disruption of BBB integrity by degradation of tight junctions in transient forebrain ischemia^29^. Although, Perls’ Prussian blue staining did not detect excessive iron accumulation in the brains of β-thalassaemia mice, it might be present at levels below the sensitivity limit. Iron could directly trigger mitochondrial dysfunction and induces oxidative stress, leading to hippocampal and cortical neuronal cell death^30,31^. In addition, neuronal loss found in β-thalassaemia mice may be caused by the activation of microglia by iron, which may further induce oxidative stress generation and neurotoxicity^32^ and initiation of neuroinflammation by secreting proinflammatory cytokines, ultimately leading to neuronal iron uptake and cell death^33^. Neuroinflammation and oxidative stress disrupt mitochondrial axonal transportation which may trigger axonal damage and transection^34^. Iron accumulation in specific in brain regions such as the hippocampus, caudate putamen, and globus pallidus is associated with cognitive dysfunction in Alzheimer's disease (AD) patients^35,36^. Moreover, a study in AD patients revealed that β-amyloid-induced oxidative stress which enhances acetylcholinesterase activity is related to cognitive impairment^37^. In β-thalassaemia mice, increased neuroinflammation and oxidative stress may cause synaptic vesicle-releasing impairment and neurotransmission failure and induce neuronal loss, contributing to cognitive impairment.

A strong correlation between cognitive impairment and anaemia markers including decreased RBC level, haemoglobin and haematocrit, was observed in β-thalassaemia mice. Anaemia can cause hypoxia and is associated with cognitive impairment in humans. Cognitive impairment has been found in chronic obstructive pulmonary disease patients and associated with hippocampal atrophy and lowered arterial blood gases (arterial oxygen tension and blood oxygen saturation)^38^. Anaemia is also associated with an increased risk of cognitive impairment, dementia, and AD^39^. Moreover, chronic hypoxia-induced cognitive impairments enhance tau hyperphosphorylation and exacerbates Alzheimer-like memory found in animal studies^40^. Remarkably, mice subjected to chronic intermittent hypoxia display increased density and morphological features of activated microglia in the dorsal hippocampus, which may be linked to the mechanism of hypoxia and neuroinflammation-indued cognitive impairment^41^.

The RBC damage was found in samples from β-thalassaemia mice, with increased PS-exposed RBCs, PS-exposed RBC large EVs and PS-exposed medium EVs, which correlated with learning and memory impairment. The increased levels of PS-exposed RBCs and PS-exposed medium EVs in β-thalassaemia patients have been reported to play a role in vasculopathy. Medium EVs could induce platelet activation and endothelial cell dysfunction, and white blood cell-endothelial cell adhesion^42,43^. In vascular dementia, impairment of cerebral blood flow, such as through atherosclerosis and stroke, can cause metabolic imbalances or structural injuries, consequently leading to cognitive decline^44^. However, due to the limited number of brain histology sections analysed, we did not observe thrombosis or haemorrhage in hippocampus area of β-thalassaemia mice.

In this study, we demonstrated that β-thalassaemia mice exhibited cognitive impairment in motor activity and in learning and memory domains, associated with intrinsic factors such as anaemia, iron overload and RBC damage. However, β-thalassaemia patients show cognitive impairment in additional domains that is beyond the scope of this investigation in a mouse model. Patients also exhibit cognitive impairment at a younger age, 19–46 years, compare to the 22-month-old β-thalassaemia mice, corresponding to 56–69 years of age in humans. Further investigation of cognitive function in younger mice is warranted. A prevalence of 26.7–60.7% of silent ischemic lesions in β-thalassaemia patients was documented by MRI^3–5^. However, our histological study found no thrombosis or haemorrhage in hippocampal area of β-thalassaemia mice. Analysis of whole brain slices in larger sample size is needed to determine the presence of thrombosis or haemorrhage in the mouse model.

As β-thalassaemia patients life expectancy is increasing, neurological complications of patients have also increased. Herein, cognitive function was investigated in 22-month-old β-thalassaemia mice to determine the effect of intrinsic factors and rule out socioeconomic effects. The β-thalassaemia mice exhibited cognitive impairment caused by iron overload, chronic anaemia and RBC damage, consequently, leading to neuronal loss in hippocampus. However, the mechanism of how iron overload and chronic anaemia contribute to cognitive impairment needs further study. Our study suggested that regular blood transfusion and iron chelator therapy may help reduce the risk of cognitive impairment. In addition, assessment of cognitive functions should become an integral part of patient follow-up to improve patients’ quality of life.

## Materials and methods

### Animals

This study was approved by the Institute of Molecular Biosciences Animal Care and Use Committee, Mahidol University (IMB-ACUC), approval number IMB-ACUC 2020/003 and IMB-ACUC 2021/020. All experiments were performed in accordance with the relevant guidelines and regulations. All experiments are reported to be in accordance with the Animal Research: Reporting of In Vivo Experiments (ARRIVE) guidelines and the American Veterinary Medical Association (AVMA) Guidelines for the Euthanasia of Animals (2020).

Twenty-two-month-old wild type C57BL/6 mice (n = 12) and β-thalassaemia mice (n = 12) were employed in the study. The β-thalassaemia mice were generated by knockout of the β-globin gene of the C57BL/6 mice^45^. Mice were housed in polystyrene cages with a room temperature of 24 ± 2°C and humidity of 55 ± 10% under a 12:12 dark–light cycle. They are fed regular chow and ultra-filtrated water ad libitum. The experimental timeline is presented in Supplementary Fig. S2. After the behavior tests, one β-thalasseamia mouse died prior to planned sacrifice.

### Analysis of phosphatidylserine exposed red blood cells and extracellular vesicles

Tail vein blood samples were collected into citrate–phosphate-dextrose-adenine (CPDA)-1 anticoagulant and immediately analysed by flow cytometry. Diluted whole blood samples were stained with fluorochrome-conjugated annexin V (PS marker, BD Biosciences, San Jose, CA)and TER119 (a RBC marker, eBioscience, San Diego, CA) in annexin V binding buffer containing TruCount beads (BD Biosciences) for absolute number calculation. The medium EV population was determined by comparison with beads with a 1-μm diameter (Spherotech, Lake Forest, IL). PS-exposed RBCs, PS-exposed RBC large EVs, PS-exposed medium EVs and PS-exposed RBC medium EVs were acquired and analysed using an Accuri C6 plus flow cytometer (BD Biosciences).

### Open field test

Open field tests were performed in a square box (50 × 50 × 50 cm). Mice (wild type n = 12; β-thalassemia n = 12) were placed in the centre of the open box and allowed to freely explore the area for 10 min^46^. The total distance traveled and speed during the trial was tracked using the S-MART video tracking software (PanLab, Barcelona, Spain*).*

### Morris water maze test (MWM)

The MWM was performed in 12 wild-type and 12 β-thalassemia mice. The MWM was performed in a 150 cm diameter circular pool filled with white colored water. The pool was divided into four quadrants (Northeast (NE), Northwest (NW), Southeast (SE) and Southwest (SW)). Four different geometric shapes were place on the pool wall, one for each quadrant to provide visual clues for the mice. A circular platform was submerged 1 cm underwater in the middle of Southwest quadrant. All swimming was tracked and analysed using the S-MART video tracking software. On the first training phase day, the visible escape platform was placed in the target quadrant at 1 cm over the water. The mouse was allowed to swim for 60 s. If the mouse could not find the visible platform, it was guided and left on the platform for 20 s. From the second to the fifth day, the platform was submerged underwater. Mice were placed in the pool facing the wall in a random quadrant and allowed to search for the escape platform for 60 s. The time and distance taken to find the platform was recorded. Each mouse received four trails per day. An hour after the last session of the fifth day, a probe trial was administered by removing the platform and recording the time spent searching in each quadrant within 60 s^47^.

### Blood collection and brain histological analysis

Mice were deeply anesthetized by i.p. injection with tiletamine-zolazepam (xylazine: 6 mg/kg; zoletil: 45 mg/kg body weight), and then, heart blood was collected into CPDA-1 anticoagulant for complete blood count and following perfusion by using 4% neutral buffered formaldehyde (NBF) solution. Brains (wild type n = 3; β-thalassemia n = 3) were removed and postfixed in 4% NBF solution at 4 °C for overnight. Then, brains were cryoprotected in 15% sucrose at 4 °C for two days and followed by 30% sucrose for 2 days followed by transfer into an OCT-filled mold, followed by rapidly freezing using isopentane chilled with dry ice. Specimens were kept into − 80 °C until cryosectioning. The 8-μm-thick coronal sections were cut using a freezing microtome (the Leica CM1950 cryostat, Leica Biosystems, IL) and sections were stained with haematoxylin and eosin for morphological analysis, Perls’ Prussian blue for tissue iron accumulation and cresyl violet acetate (Nissl staining) for living and dark neuron analysis. Eight different fields (4 continuous field of each left and right brain, respectively) of primary motor cortex and 10 different fields (5 continuous field of each left and right brain, respectively) of hippocampus were captured using light microscope (Olympus CX33 HD Digital Microscope Package, Canoscope 5 MP FHD digital camera, model DG-105-W, CaptaVision Imaging Software, Japan). The number of living and dark neuronal cells was calculated by Image J software (National Institutes of Health, Bethesda, MD) (Supplementary Figs. S3–S7).

### Liver tissue iron analysis

The liver iron content of 12 wild-type and 12 β-thalassemia mice was evaluated by a colorimetric method^48^. Briefly, non-haeme iron in freeze-dried liver tissue was extracted with 3 M HCl and reduction with 10% trichloroacetic acid, and then, examined by adding a chromogen reagent (0.1% bathophenanthroline sulfonate and 1% thioglycolic acid) and measuring the optical density at 535 nm. The iron concentration was then determined based on an iron standard curve.

### Statistical analysis

Data were analysed using SPSS Version 18.0 (IBM Collaboration, Chicago, IL) and GraphPad PRISM 6.0 (Graph-Pad Software, San Diego, CA). Comparisons between parameters including open field test, MWM test and number of neuron cells were evaluated with the T-test. Comparisons between parameters including complete blood count, liver tissue iron, damaged RBCs and EVs were evaluated with a non-parametric Mann–Whitney U test. The correlation coefficient was calculated with Spearman’s rho (r_s_). The threshold for statistical significance for all comparisons was *P* < 0.05.

### Supplementary Information

Supplementary Figures.

## Data Availability

All data related to this study can be obtained on request, while all the analyzed data are included in this published article and its supplementary information files.
