# Supplementary material for: Cognitive impairment and hippocampal neuronal damage in β-thalassaemia mice
Source: Sci Rep. 2024 May 2;14:10054. doi: 10.1038/s41598-024-60459-y (PMC11066061; doi:10.1038/s41598-024-60459-y)
Supplement: Supplementary file 1 — Supplementary Figures. [file 41598_2024_60459_MOESM1_ESM.pdf]

Supplementary information

Cognitive impairment and hippocampal neuronal damage in β-thalassaemia mice

Nuttanan Pholngam[1,2], Parinda Jamrus[2,3], Kittikun Viwatpinyo[4,5], Benjaporn Kiatpakdee[2], Jim Vadolas[6,7], Pornthip Chaichompoo[3], Sukonthar Ngampramuan[4,*], Saovaros Svasti[2,8,*]

[1]Graduate Program in Molecular Medicine, Faculty of Science, Mahidol University, Bangkok, Thailand; [2]Thalassemia Research Center, Institute of Molecular Biosciences, Mahidol University, Nakhon Pathom, Thailand; [3]Department of Pathobiology, Faculty of Science, Mahidol University, Bangkok, Thailand; [4]Research Center for Neuroscience, Institute of Molecular Biosciences, Mahidol University, Nakhon Pathom, Thailand; [5]Department of Medical Science, School of Medicine, Walailak University, Nakhonsithammarat;[6]Centre for Cancer Research, Hudson Institute of Medical Research, Melbourne, Australia; [7]Department of Molecular and Translational Science, Monash University, Melbourne, Australia; [8]Department of Biochemistry, Faculty of Science, Mahidol University, Bangkok, Thailand.

***Correspondence:**

Saovaros Svasti, Ph.D., Thalassemia Research Center, Institute of Molecular Biosciences, Mahidol University, Nakhon Pathom 73170 Thailand. Phone: +662-889-2558; Fax: +662-889-2559; e-mail: saovaros.sva@mahidol.ac.th, stssv@yahoo.com

Sukonthar Ngampramuan, Ph.D., Research Center for Neuroscience, Institute of Molecular Biosciences, Mahidol University, Nakhon Pathom 73170 Thailand. Phone: +662-441-9003-7

(1206, 1311); Fax: +662-441-1013; e-mail: sukonthar.nga@mahidol.edu, sukonthar.nga@ mahidol.ac.th

Number of supplementary figure: 7

Supplementary figures

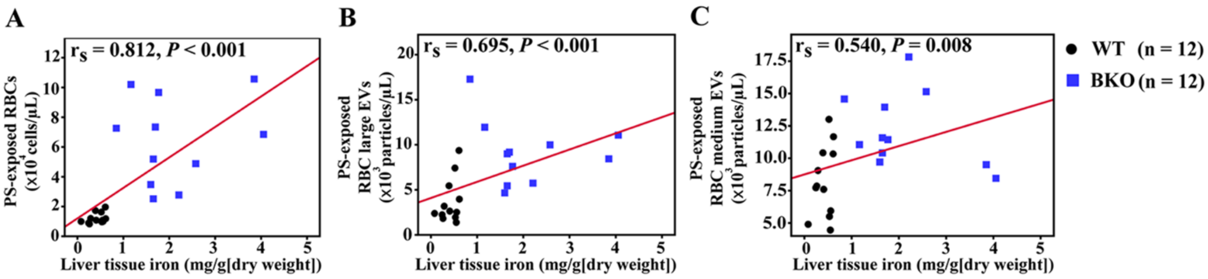

Figure S1 Spearman's rho coefficient correlation between liver tissue iron and (A) PS-exposed RBCs, (B) PS-exposed RBC large EVs and (C) PS-exposed RBC medium EVs. BKO; β-thalassaemia mice, EVs; extracellular vesicles, RBCs; red blood cells, PS; phosphatidylserine, WT; wild type mice.

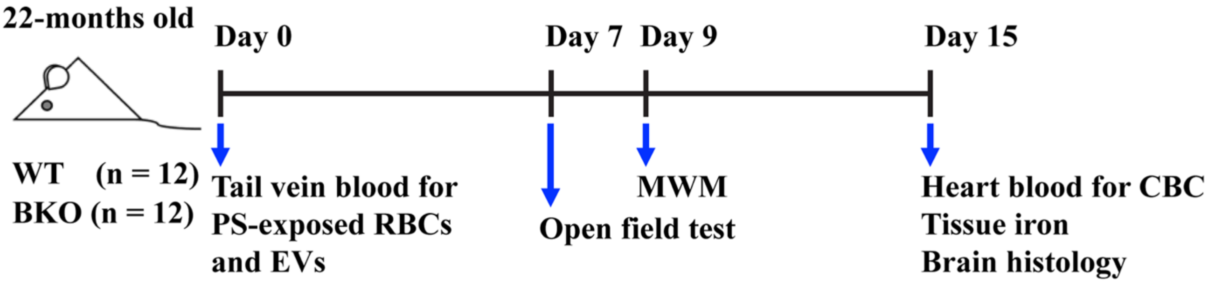

Figure S2 Timeline of mouse sample collection and behavioral experiments. Tail vein blood samples were collected at 22-months old from wild type (WT) and β-thalassaemia (BKO) mice for flow cytometric analysis. The mice were allowed wound to heal for 7 days before performed open field tests and Morris water maze (MWM) tests. Heart blood, liver and brain samples were collected at day 15 for complete blood count (CBC), tissue iron and histological analysis, respectively.

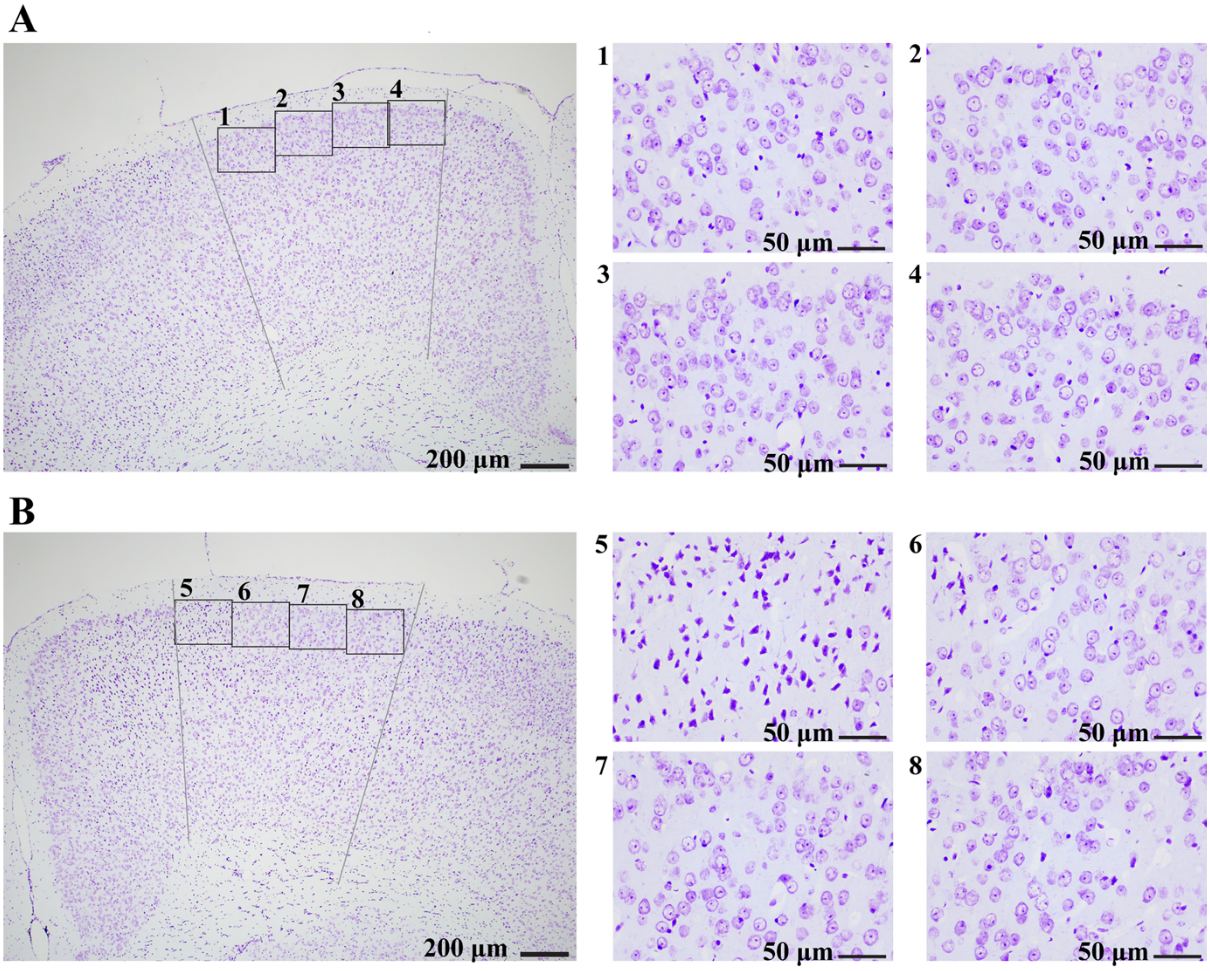

Figure S3 Primary motor cortex, layer 2/3 area. Brain from β-thalassaemia mouse was cryoprotected and cryosection at 8-µm-thick coronal section. Brain section was performed Nissl stain and captured total area of primary motor cortex, layer 2/3 from (A) left- and (B) right-side at 40× magnification that was divided the individual side into 4 continuous fields (left-side; number 1-4, right-side; number 5-8, respectively) at 400× magnification by using light microscope (Olympus CX33 HD Digital Microscope Package, Canoscope 5 MP FHD digital camera, model DG-105-W, CaptaVision Imaging Software, Japan).

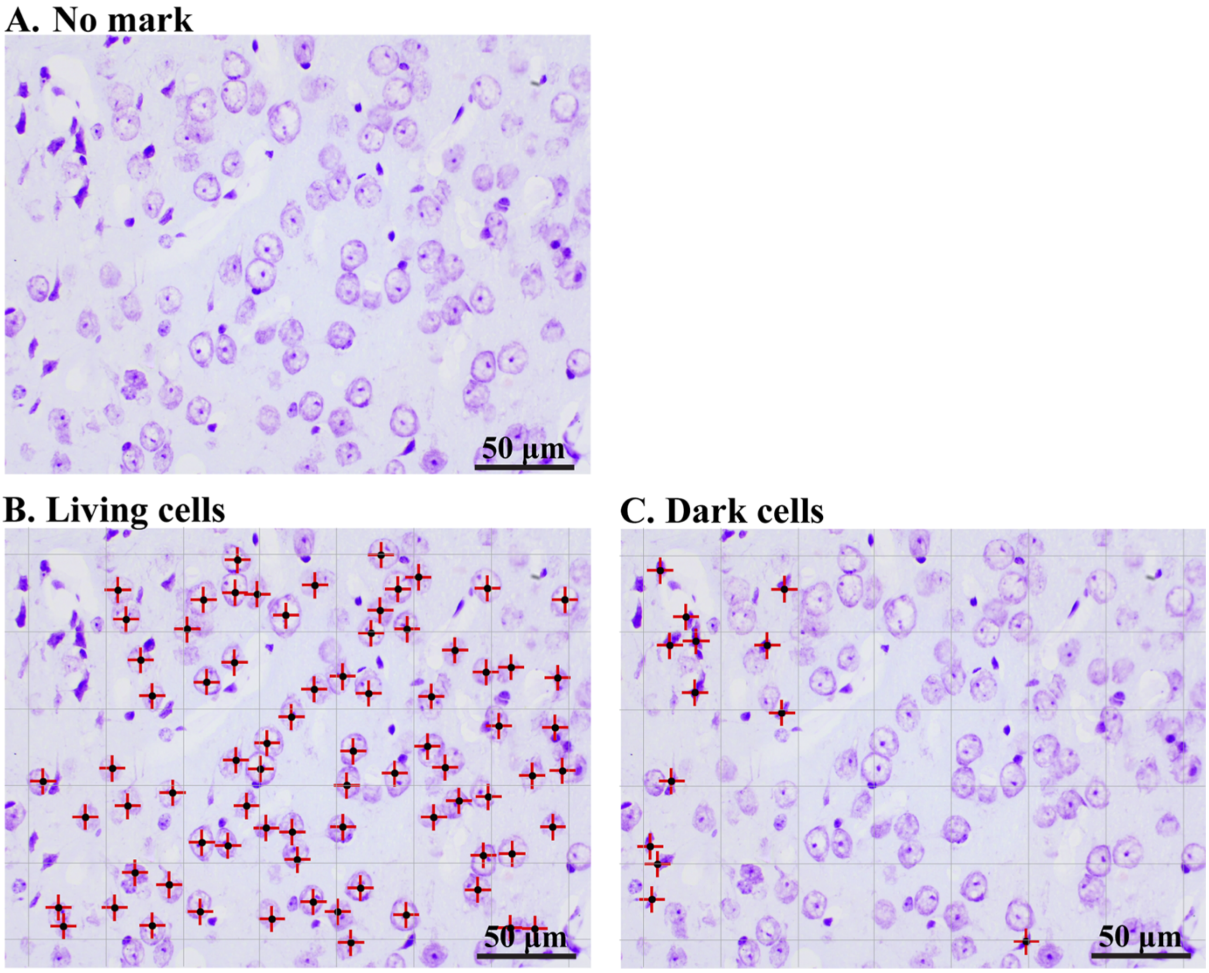

Figure S4 Morphological identification of living and dark neuron in primary motor cortex, layer 2/3 area. (A) Nissl-stained brain from β-thalassaemia mouse was captured at 400× magnification by using light microscope (Olympus CX33 HD Digital Microscope Package, Canoscope 5 MP FHD digital camera, model DG-105-W, CaptaVision Imaging Software, Japan). Illustration was analyzed the total investigated area and count (B) living neuron cells and (C) dark neuron cells using Image J software (National Institutes of Health, USA). The black dot with red mark is counting the number of cells.

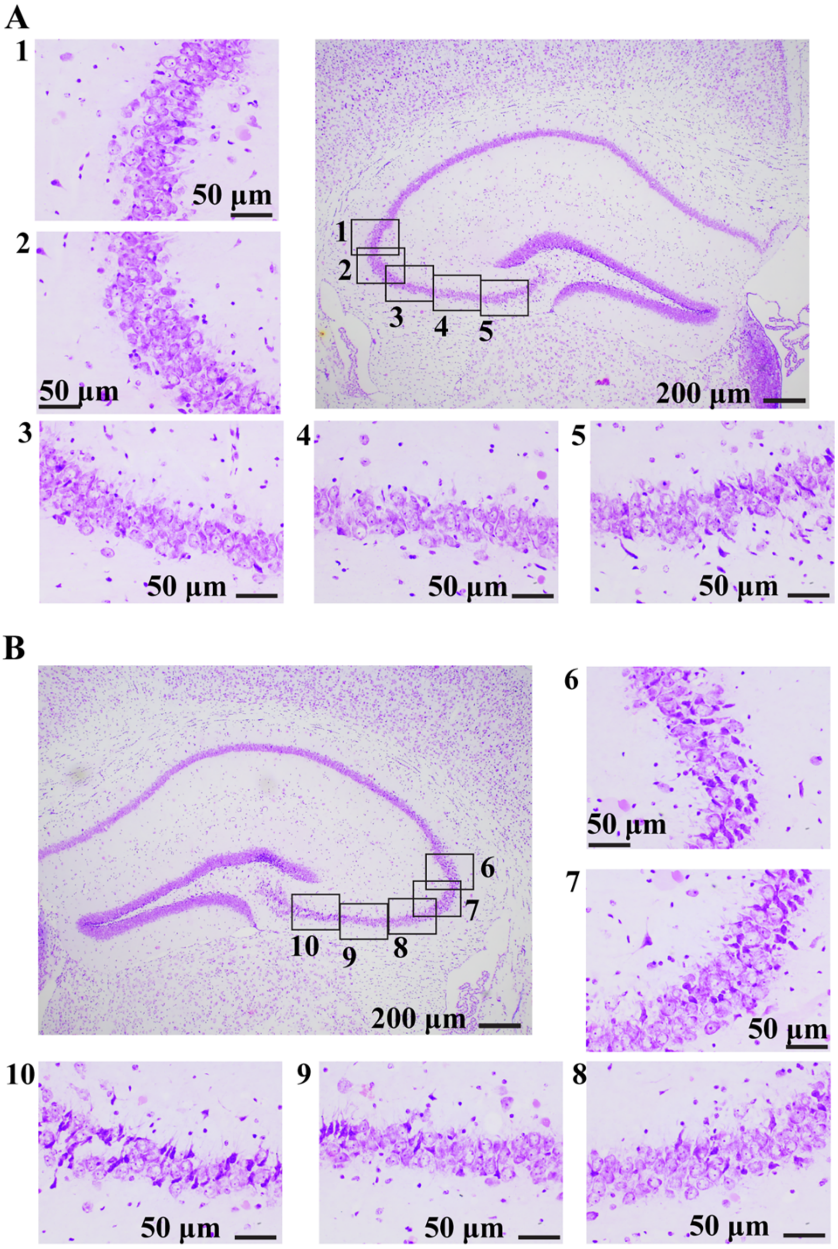

Figure S5 Hippocampus, CA3 area. Brain from β-thalassaemia mouse was cryoprotected and cryosection at 8-µm-thick coronal section. Brain section was performed Nissl stain and captured total area of hippocampus at CA3 layer from (A) left- and (B) right-side at 40× magnification that was divided the individual side into 5 continuous fields (left-side; number 1-5, right-side; number 6-10, respectively) at 400× magnification by using light microscope (Olympus CX33 HD Digital Microscope Package, Canoscope 5 MP FHD digital camera, model DG-105-W, CaptaVision Imaging Software, Japan).

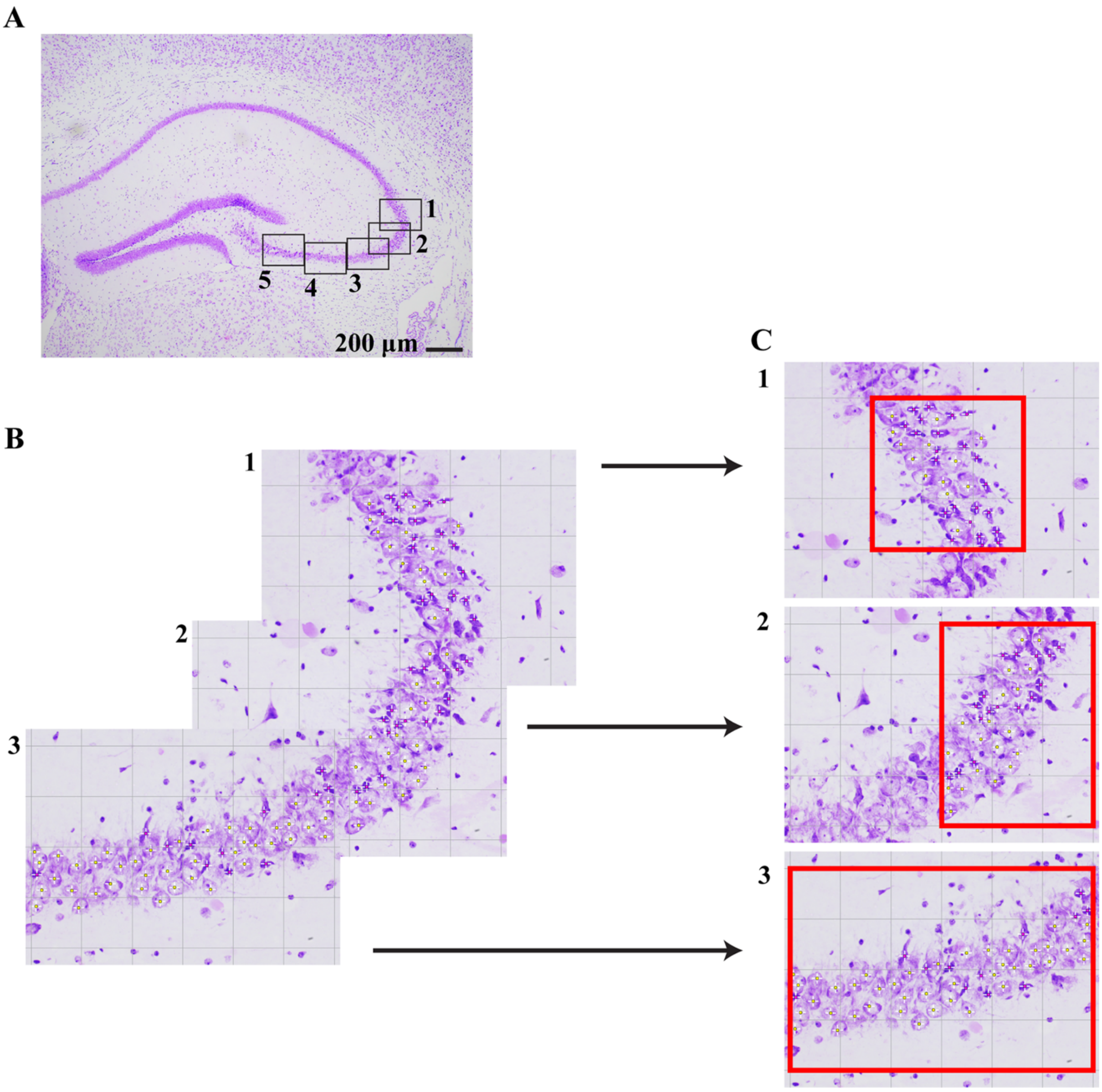

Figure S6 Continuing area using grid analysis. (A) Nissl-stained brain from β-thalassaemia mouse was observed CA3 region of hippocampus by divided into 5 fields: number 1-5. Hippocampus at CA3 area is C-shaped structure as Ammon's horn, especially field number 1-3 shown the overlap areas. (B-C) Therefore, the individual illustration was grided for count living neuron cells (yellow mark) and dark neuron cells (purple mark) in the continuing area to avoid the repeated or missed count the cells.

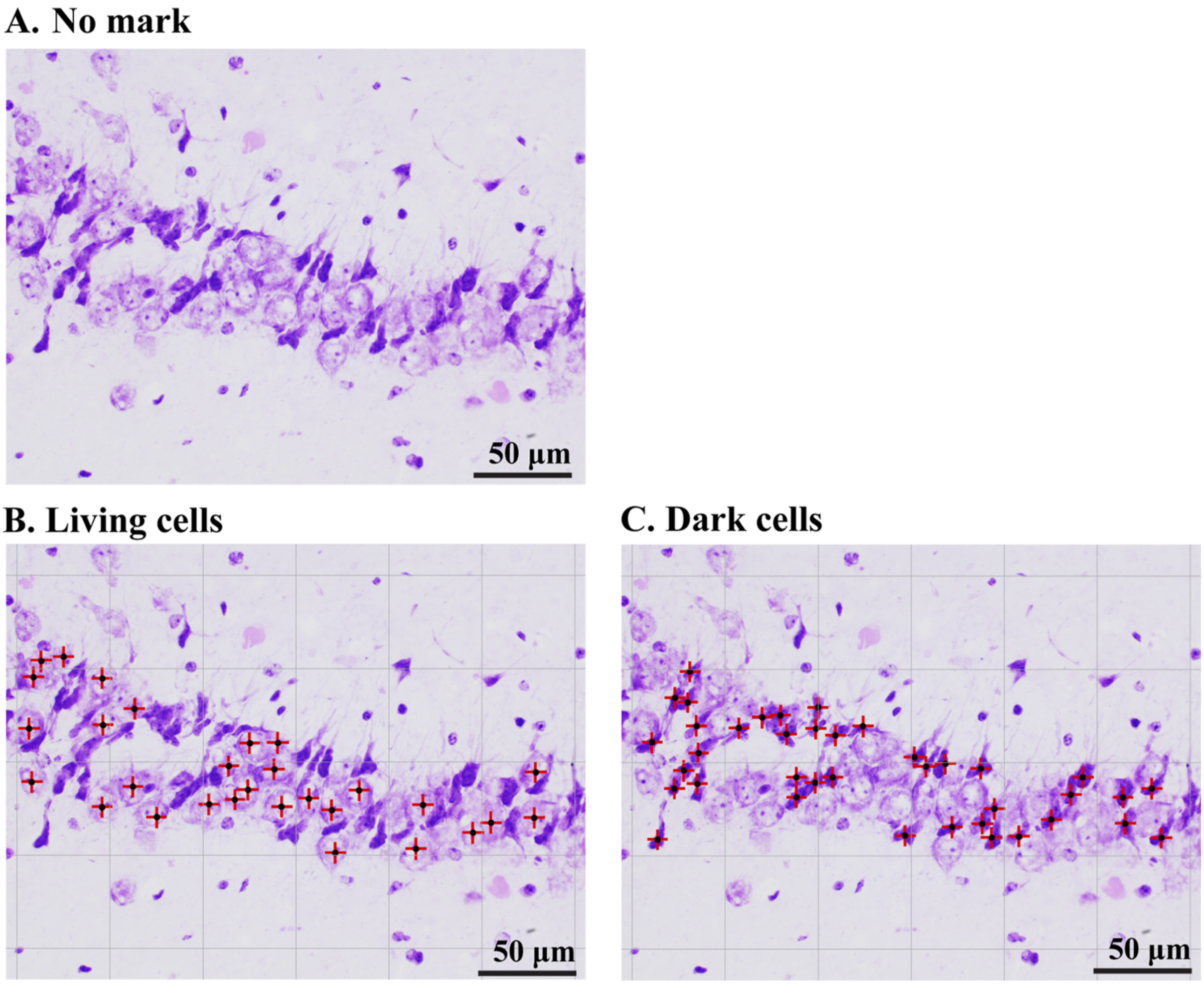

Figure S7 Morphological identification of living and dark neuron in hippocampus, CA3 area. (A) Nissl-stained brain from β-thalassaemia mouse was captured at 400× magnification by using light microscope (Olympus CX33 HD Digital Microscope Package, Canoscope 5 MP FHD digital camera, model DG-105-W, CaptaVision Imaging Software, Japan). Illustration was analyzed the total investigated area and count (B) living neuron cells and (C) dark neuron cells using Image J software (National Institutes of Health, Bethesda, MD). The black dot with red mark is counting the number of cells.
